# The Entanglement between Mitochondrial DNA and Tumor Metastasis

**DOI:** 10.3390/cancers14081862

**Published:** 2022-04-07

**Authors:** Qiwei Wu, Hsiang-i Tsai, Haitao Zhu, Dongqing Wang

**Affiliations:** 1 Department of Medical Imaging, The Affiliated Hospital of Jiangsu University, Zhenjiang 212001, China; 2211913036@stmail.ujs.edu.cn; 2 Laboratory of Radiology, The Affiliated Hospital of Jiangsu University, Zhenjiang 212001, China; tsaihsiangi88@163.com

**Keywords:** mitochondrial DNA, tumor progression, metastasis, immune escape

## Abstract

**Simple Summary:**

Mitochondrial dysfunction is one of the main features of cancer cells. As genetic material in mitochondria, mitochondrial DNA (mtDNA) variations and dysregulation of mitochondria-encoded genes have been shown to correlate with survival outcomes in cancer patients. Cancer metastasis is often a major cause of treatment failure, which is a multi-step cascade process. With the development of gene sequencing and in vivo modeling technology, the role of mtDNA in cancer metastasis has been continuously explored. Our review systematically provides a summary of the multiple roles of mtDNA in cancer metastasis and presents the broad prospects for mtDNA in cancer prediction and therapy.

**Abstract:**

Mitochondrial DNA, the genetic material in mitochondria, encodes essential oxidative phosphorylation proteins and plays an important role in mitochondrial respiration and energy transfer. With the development of genome sequencing and the emergence of novel in vivo modeling techniques, the role of mtDNA in cancer biology is gaining more attention. Abnormalities of mtDNA result in not only mitochondrial dysfunction of the the cancer cells and malignant behaviors, but regulation of the tumor microenvironment, which becomes more aggressive. Here, we review the recent progress in the regulation of cancer metastasis using mtDNA and the underlying mechanisms, which may identify opportunities for finding novel cancer prediction and therapeutic targets.

## 1. Introduction

Tumor metastasis accounts for the overwhelming number of deaths in cancer patients. Tumor metastasis is a multi-step process [1], including the invasion, migration and adhesion of tumor cells; immune escape; and repopulation in the second location. An increasing number of underlying molecular mechanisms that are involved in tumor metastasis progression are being revealed. Among these factors, the role of mitochondrial DNA (mtDNA) and the related mitochondrial dysfunction have attracted significant attention.

From tumorigenesis to metastasis, cancer cells depend on metabolic reprogramming to survive [2]. Warburg proposed that unlike normal cells, cancer cells do not need to rely on the oxidative phosphorylation (OXPHOS) system to generate ATP. Even under aerobic conditions, cancer cells can convert glucose into lactic acid through glycolysis and produce ATP [3]. We call this theory the Warburg effect or aerobic glycolysis, which has become a biochemical marker of cancer. The unlimited proliferation ability of cancer cells results in their craving for ATP and metabolites. Mitochondria are crucial for oxidizing glucose, fats and amino acids to release energy through the tricarboxylic acid (TCA) cycle and OXPHOS [4], which is thought to be the “energy factory” of cancer cells [5]. The transfer mitochondria from aggressively growing tumors to less aggressive tumors can cause an increase in tumor aggressiveness, indicating the critical role of mitochondria in determining the cancer cell biology [6]. With the in-depth study of the mitochondrial genome, more evidence has revealed the relationship between mtDNA abnormalities and many diseases, especially cancer. Moreover, the alterations of genes encoding mitochondrial components are associated with increased cancer risk, and may even become carcinogenic factors [7].

As the core component of mitochondrial, mtDNA encodes 13 OXPHOS-system-related protein complexes (complex I, III-V) subunits [8], which drives mitochondrial respiration and energy production [9]. Mitochondrial dysfunction has previously been reported to enhance the tumorigenicity and metastatic potential of lung and breast cancer cells [10,11,12]. Mutations in mtDNA (for example, insertion of the MT-ND6 gene) can disrupt the function of complex I, leading to an increase in ROS, while the corresponding tendency for metastasis is also increased [10,13]. This obstacle also leads to the upregulation of glycolysis and metastasis-related gene transcription [13]. The altered function of complex I in metastasis was subsequently reconfirmed in the MDA-MB-231 breast cancer cell line, and its increased metastatic potential appeared to be associated with mutations in MT-ND6 (C12084T) and MT-ND5 (A13966G). The missense and nonsense mutations in MT-ND6 enhanced tumor invasion and migration, as also demonstrated in the A549 lung cancer cell line [14]. Therefore, mtDNA is an important factor in tumor initiation and progression [6,15,16].

As more in-depth studies of mitochondrial-encoded factors emerge, this may lead to exciting advances in targeting mtDNA to inhibit tumor metastasis. This review summarizes the structure and function of the mitochondrial genome and the relationship between mtDNA and tumor metastasis. Our goal is to establish a framework in related fields and address the role of the mitochondrial genome in tumor metastasis.

## 2. mtDNA

### 2.1. Structures of mtDNA

**Figure 1:**
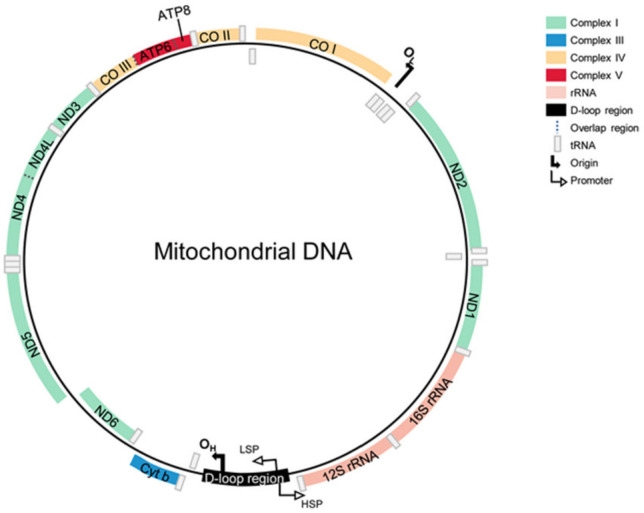
The structure of mtDNA. It encodes 2 rRNAs and 22 tRNAs for protein synthesis, as well as 13 peptides for electron transfer and oxidative phosphorylation. Except for these coding areas, mtDNA has a non-coding region located in the displacement (D)-loop, which participates in the regulation of mtDNA replication transcription. The genes coding for subunits of OXPHOS complex I are ND1-ND6. The gene encoding for cytochrome B of complex III is abbreviated as Cyt B. Genes for cytochrome c oxidase (complex IV) are CO I-CO III. Additionally, the subunits of complex V are ATPase 6 and 8, abbreviated as A6 and A8, respectively. The two ribosomal RNAs encoded by mtDNA are 12S and 16S. Except for these coding areas, mtDNA has a non-coding region located in the displacement (D)-loop, which participates in the regulation of mtDNA replication transcription. The displacement loop is represented as the D-loop and contains sequences for the initiation of replication and transcription, including the origin of heavy-strand replication (O_H_). The light-strand replication’s origin is indicated by O_L_. The position of the light-strand promoter is shown as LSP and the position of the heavy-strand promotor as HSP.

The special form of deoxyribonucleic acid is mtDNA. It is the only genetic material outside the nucleus that can be copied, transcribed and translated independently [17]. Human mtDNA is a circular-shaped, double-stranded DNA measuring about 16,569 bp. The mtDNA includes a G-enriched inner light strand (L-strand) and a C-enriched heavy strand (H-strand) [9,18]. Human mtDNA is a genetically compact genome with no introns, which overlaps in some regions. It encodes 2 rRNAs (16S RNA, 12S RNA) and 22 tRNAs for protein synthesis, as well as 13 peptides for electron transfer and oxidative phosphorylation. In addition to these coding areas, mtDNA also consists of a non-coding region, the displacement (D)-loop, which participates in the regulation of mtDNA replication transcription [19] (Figure 1).

### 2.2. Characteristics and Maintenance of mtDNA

Almost every base of mtDNA is involved in gene construction, meaning any mutation would affect an important functional region of the genome [9]. Furthermore, mtDNA has its own unique biological environment and properties: (1) The mtDNA is vulnerable to ROS damage due to its lack of histone protection [20,21]. (2) The mtDNA has a high mutation rate [22,23], which may be due to its relatively naive protection and repair mechanisms. Currently, mtDNA damage can usually only be repaired via base excision repair (BER), homologous recombination (HR) and microhomology-mediated end joining (MMEJ) [24]. (3) Due to its small molecular mass and the absence of introns, mtDNA shows continuous synthesis throughout the cell cycle, although this is not synchronized with the cell cycle and mainly occurs in the S and G2 phases, which makes the dynamic process more susceptible to interference by external factors. (4) It has a high copy number. In many cells, the volume of mtDNA is higher than needed to maintain oxidative phosphorylation. Furthermore, the poor proofreading ability of DNA polymerase and the easy formation of a hairpin structure in the RNA transfer position results in the mtDNA replication process being more prone to mistakes. Although its replication is independent of nDNA replication, the trans-acting factors, copy number and integrity of mtDNA are closely regulated and encoded by nDNA. The nDNA-encoded polymerase g (POLG) and mitochondrial transposition factor A (TFAM) [25] are necessary to maintain the copy number and integrity of mtDNA [26,27,28]. Moreover, these proteins bind to the D-loop region of mtDNA, forming NUCLEOIDS, which are considered to be units of mtDNA transmission and inheritance [29]. TFAM is also the main component that initiates and drives mtDNA packaging and the NUCLEOIDS structure, and is directly proportional to the mtDNA copy number [30]. Therefore, mtDNA could bind to form complexes with TFAM rather than being naked as previously thought [29,31].

The mtDNA copy number may also have an unknown regulatory mechanism. As mitochondria divide randomly in daughter cells, there may be two conditions during cell division: homoplasmy (the same genotype) and heteroplasmy (coexistence of wild-type and mutant mitochondrial genomes) [32]. Normally, all mtDNA in a cell should be identical. In heterogeneous cells, owing to the different mitochondrial genomes in each cell, the ratio of mutants to wild-type mitochondria determines whether the cell is deficient of energy; that is, when the mutation reaches a certain ratio, an impaired phenotype occurs and lead to diseases, which is called the threshold effect [33]. The threshold level is dependent on the tissue type, metabolic requirements and copy number of remaining WT mtDNA (aberrant mitochondrial function in aging and cancer). It has also been shown that a decrease in mtDNA replication machinery or enhanced mitophagy results in a lower mtDNA copy number [34]. Meanwhile, doxorubicin (Dox)-induced mitochondrial phagocytosis in cardiomyocytes resulted in a decrease in the copy number of mitochondrial DNA [35]. This suggests that mitophagy is quite important in mtDNA copy number regulation, which needs to be demonstrated by further studies. As a multi-genome with strict control over the copy number, the regulatory mechanism of the mtDNA copy number remains unclear. It seems to be controlled by cell-specific mechanisms and regulated by various internal environmental stressors.

### 2.3. The Release of mtDNA

Mitochondrial dynamics play an important role in the nuclear distribution of mtDNA, cristae reorganization and the mitochondrial pro-apoptotic state [36]. The deletion of mitochondrial TFAM significantly altered the packaging and distribution of mtDNA and induced mtDNA release into the cytoplasm, which was considered to be “cytoplasmic mtDNA stress” [37]. Furthermore, studies have shown that mtDNA can be released from apoptotic mitochondria. BAX/BAK is activated during apoptosis, whereby large BAX/BAK pores appear on the outer membrane of mitochondria, while the inner membrane of mitochondria can herniate into the cytoplasm and carry mtDNA [38,39]. Mitochondrial membrane damage and mitochondrial division can also cause the release of mtDNA into the cytoplasm. When the mitochondria are subjected to stress in a variety of ways, mtDNA breaks into fragments and then binds to the voltage-dependent anion channel (VDAC) in the outer membrane of the mitochondria. This leads to the aggregation of multiple VDAC monomers and the formation of a pore in the middle through which mtDNA can escape [40]. In conclusion, changes in mitochondrial morphology and dynamics can promote the release of mtDNA. The release of mtDNA and its vulnerability to damage have been found to be involved in tumorigenesis and progression.

## 3. mtDNA and Tumor Metastasis

**Figure 2:**
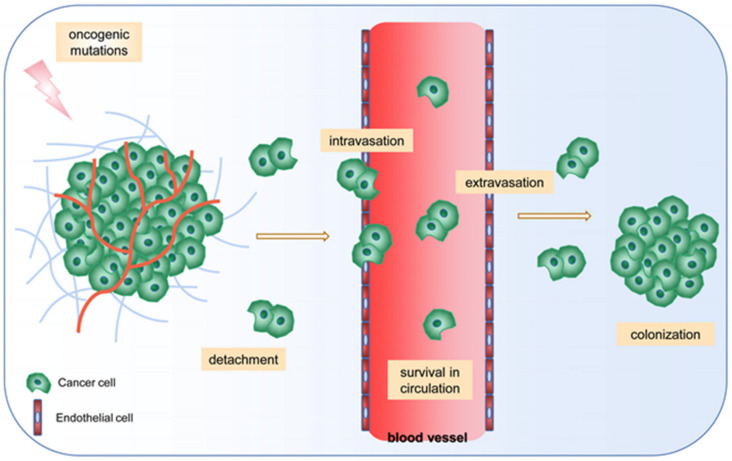
Steps of tumor metastasis. The process of tumor metastasis mainly goes through five stages: (1) local detachment of tumor cells and invasion of surrounding tissues (resistance to anoikis); (2) intravasation, where tumor cells enter the blood or lymphatic system; (3) tumor cell transport and survival in the circulatory system; (4) exudate leakage from circulation into distal tissues; (5) colonization, to form metastatic foci. Obstruction of any of these steps would prevent the formation of the metastasis. As early as 1889, Stephen Paget first proposed the seed and soil hypothesis, which has been widely recognized as the critical theory for tumor metastasis.

Tumor metastasis is a series of invasive and metastatic cascades in which tumor cells detonate from the original tumor to reach surrounding or distal tissues to form new lesions. It is the result of the interaction between tumor cells and the tumor microenvironment [41]. Metastasis is the main cause of death in tumor patients, with most patients dying from the spread of the cancer rather than the primary tumor [42]. The process of tumor metastasis mainly goes through five stages: (1) local detachment of tumor cells and invasion of surrounding tissues (resistance to anoikis) (anoilis: loss of cell adhesion to the ECM and lead to apoptosis); (2) intravasation, where tumor cells enter the blood or lymphatic system; (3) tumor cell transport and survival in the circulatory system; (4) exudate leakage from circulation into distal tissues; (5) colonization, to form metastatic foci. Obstruction of any of these steps would prevent metastasis (Figure 2).

To date, there is no clear evidence of a definitive relationship between mtDNA and tumor metastasis, but some non-random changes in the mitochondrial genome associated with tumor progression have been reported in many tumor types [14,43,44]. We reviewed the literature on mtDNA alterations that cause or are associated with metastatic mutations, and there is considerable evidence that mtDNA alterations can accelerate tumor progression by enhancing the invasiveness and metastatic potential of tumor cells. Studies have supposed that replacing mtDNA from poorly metastatic tumor cells with mtDNA from highly metastatic tumor cells in mice via cytoplasmic hybrid (cybrid) technology could result in better metastatic capacity [45]. Thus, mtDNA alterations have an important role in enhancing a tumor’s metastatic capacity. This was consistent with the conclusion by Amanda E. Brinker [46]. They used a novel mouse model, a mitochondrial nuclear exchange model termed MNX, to verify whether the mitochondrial genome affects the tumor incubation period and metastasis efficiency. The mitochondrial haplotype altered the tumorigenicity and metastasis of breast cancer in an oncogenic driver-dependent manner [46]. As expected, there was an inherently strong relationship between mtDNA and tumor metastasis. Here, we summarize the role played by mtDNA in the various stages of tumor metastasis.

### 3.1. mtDNA during Cancer Cell Anti-Anoikis

Metastasis is not caused by the random survival of cells shed by the primary tumors, but by the selective growth of specialized subsets of highly metastatic cells [47]. Metastatic cells can be generated through clonal evolution or clonal selection, and mutational genetic drivers within tumor cells confer proliferative and invasive properties [48]. Loss of cell adhesion to the ECM leads to apoptosis, called anoikis. Resistance to anoikis is a prerequisite for tumor metastasis. Deletion of mtDNA in prostate epithelial PNT1A cells was demonstrated to prevent anoikis and promote the migratory capacity of basement membrane proteins via the upregulation of p85 and p110 phosphatidylinositol 3-kinase (PI3K) subunits [49]. Inhibition of PI3K, siRNA-mediated Akt2 depletion and mtDNA reconstitution were sufficient to restore the sensitivity of tumor cells to anoikis and reduce their migration. In addition, Akt2 activation induced glucose transporter 1 (GLUT1) expression, glucose uptake and lactate production, which is a common phenotypic change in tumor cells [49]. Unlike normal cells, due to the Warburg effect, tumor cells are resistant to anoikis. Tumor cells show restricted OXPHOS and ROS before detachment [50]. Pyruvate dehydrogenase kinase (PDK) is an important mitochondrial enzyme in glucose metabolism. High expression of PDK can block OXPHOS, promote the Warburg effect [51] and mitigate excessive ROS produced by glucose oxidation. To some extent, it protects tumor cells from ROS-induced anoikis to promote metastasis [52].

In addition, cancer stem cells (CSCs) present in the primary tumor can be resistant to anoikis and have an inherent tendency to metastasize. Both clonal selection and CSCs may synergistically produce cells with metastatic ability. In breast cancer, a decrease in mtDNA can promote the production of breast cancer stem cells, thereby promoting breast cancer metastasis. Similarly, mtDNA deficiency may induce stem-cell-like properties in ovarian cancer in different ways in vitro, leading to different tumor behavior [53]. Compared to non-cancerous tissues, the mtDNA copy number was shown to be relatively reduced in lung, gastric, breast, renal cell, colorectal and prostate cancers, while mtDNA oxidative damage was increased [54,55,56,57,58,59]. A decreased mtDNA copy number was also found to be associated with metastasis to Ewing’s sarcoma [60] and shorter survival in astrocytoma patients [61]. The mutations of mtDNA may be accompanied by decreased respiratory enzyme complex activity and mitochondrial dysfunction, leading to a malignant phenotype of the tumor [62]. It is interesting that the increased mtDNA copy number has also been reported to be associated with increased malignancy of tumors. The mtDNA copy number was increased in pancreatic, head and neck, esophageal and other cancers [63,64,65]. In these tumors, a higher mtDNA copy number indicated higher biosynthetic activity and energy activity, which was beneficial for cancer cells to achieve efficient adaption to the microenvironment. Furthermore, mitochondrial dysfunction caused by mtDNA mutations was also shown to promote the cell remodeling of cancer cells. The acquisition of mtDNA from host cells partially restored mitochondrial function in tumor cells, reestablishing the respiration to promote tumor growth. Lung metastatic tumor cells then exhibited complete restoration of respiratory function. These results indicated that the process of mtDNA transfer from host cells in the tumor microenvironment to tumor cells with impaired respiratory function overcame the pathophysiological process of mtDNA damage and supported the high plasticity of tumor cells [66].

### 3.2. mtDNA during Cancer Cell Intravasation or Extravasation

**Figure 3:**
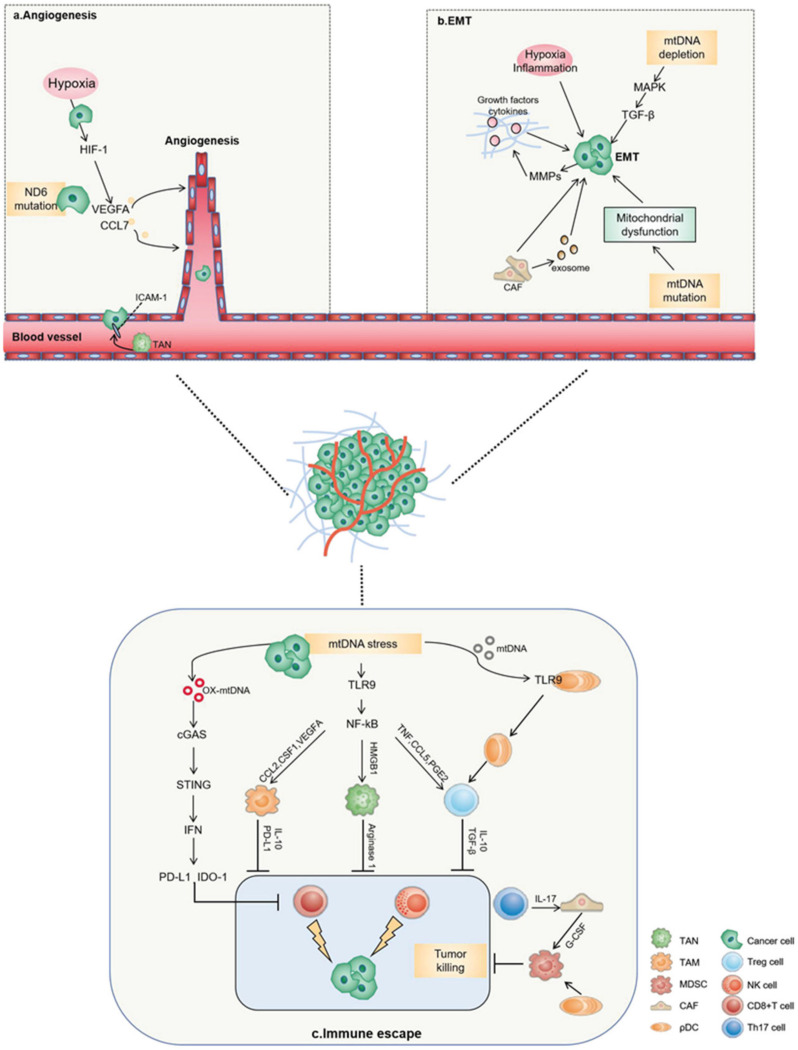
The association between mtDNA and tumor metastasis. (**a**) Angiogenesis is a rate-limiting factor in tumor growth. Cancer cells carrying the 13885insC mutation of the ND6 subunit have higher spontaneous metastasis potential. A PCR array analysis showed higher levels of VEGF, CCL7 and other metastasis-associated genes. The increase in VEGF and CCL7 levels is obviously an important factor in stimulating tumor blood vessel growth to promote tumor cell invasion, migration and infiltration. (**b**) EMT is a process in which the epithelial tumor cells lose adhesion ability and cause the migration of mesenchymal cells to promote metastasis and drug resistance. The mtDNA depletion can lead to a loss of characteristic epithelial cell features and to a mesenchymal phenotype being gained. The RAF/MAPK signaling pathway is highly activated and accompanied by the expression of TGF-β. The above suggest that the tumor cells acquire higher invasiveness. The mutations of mtDNA may be accompanied by decreased respiratory enzyme complex activity and mitochondrial dysfunction, which leads to the malignant phenotype of the tumor. (**c**) Tumor immune escape is a critical step in tumor metastasis and malignant progression. Oxidized mtDNA can be released into the cytoplasm, which induces IFN signals through cGAS-STING-TBK1, thereby upregulating the expression of PD-L1 and IDO-1 to inhibit the activation of T cells. It has been reported that mtDNA stress in HCC cells with increased mitochondrial division significantly increases CCL2 production by activating the TLR9-mediated NF-κB signaling pathway, thereby promoting TAM recruitment and polarization and consequently promoting tumor progression. Mitochondrial DAMP (including mtDNA) can activate neutrophils to generate NETs, which have been shown to promote cancer-related formation and are associated with accelerated metastasis. Immature pDC can recognize the CpG sequence on mtDNA through TLR9, and can induce the production of CD4^+^CD25^+^Treg cells after activation and maturation. Treg cells secrete cytokines IL10 and TGF-β, which produce powerful immunosuppressive functions.

The rapid proliferation of tumor cells leads to tumor ischemia and hypoxia, which directly stimulates angiogenesis and promotes the secretion of cytokines such as matrix metalloproteinase-9 (MMP9), C-X-C motif chemokine ligand 12 (CXCL12) and Wnt7B by various cells, especially tumor cells. These cytokines also promote the proliferation of endothelial cells, angiogenesis and an increase in vascular permeability [67]. The neoplastic endothelial cells express high-adhesion molecules and connect to the original vascular endothelial cells, allowing continued vascular extension. In the initial stage of tumor metastasis, tumors locally infiltrate into the vascular or lymphatic system. Tumor cells can be activated by inflammatory signals and invade the matrix by secreting proteases that degrade extracellular matrix proteins. The tumor cells enter the neovascularization and then metastatic spread occurs with the extension of the vessels. Lung cancer cells carrying the 13885insC mutation of the mtDNA ND6 subunit had a higher spontaneous metastasis potential [13]. A PCR array analysis revealed a higher level of vascular endothelial growth factor (VEGF), CCL7 and other metastasis-associated genes. The increase in VEGF levels was obviously an important factor in stimulating tumor blood vessel growth. CCL7 was shown to recruit monocytes to promote tumor cell invasion, migration and infiltration by stimulating tumor angiogenesis. In primary tumors, tumor cells produced VEGF and fibroblast growth factor 1 (FGF1) to increase vascular density. Interactions with endothelial cells through VEGF and matrix metalloproteinase-1 (MMP1) signaling pathways enhanced vascular permeability, which provided favorable conditions for tumor metastasis [68]. During the process of extravasation, tumor cells could initiate metastasis by adhering to endothelial cells through various adhesion factors, while neutrophils could enhance tumor cell adhesion and transendothelial migration through the integrin–intercellular adhesion molecule-1 (ICAM-1) signaling pathway [69]. In ovarian cancer cells with a reduced mtDNA copy number, the upregulation of gene expression related to tumor metastasis and angiogenesis more powerfully confirmed the role of mtDNA in promoting tumor angiogenesis [53]. Neonatal tumor vessels provided essential oxygen and nutrients for tumor growth and metastasis. In contrast to normal vessels, tumor vessels were characterized by discontinuous pericyte coverage, an incomplete vascular matrix and high permeability [70] (Figure 3a). It has been reported that endogenous bacterial endotoxin lipopolysaccharides in sepsis can activate Gasdermin D and form mitochondrial pores to release mtDNA into the cytoplasm, which may cause the activation of the cGAS-STING pathway and suppress YAP signaling, thereby inhibiting the proliferation of endothelial cells [71]. Endothelial cells play a significant role in tumor metastasis. It is speculated that as a DAMP, mtDNA can promote tumor metastasis by inhibiting endothelial cell proliferation and mediating endothelial cell dysfunction in tumor cells.

Endothelial cells and pericytes constitute the physical barrier of tumor vessels, but some pericytes could be activated or undergo pericyte fibroblast transformation, leading to the loss of barrier function to promote tumor metastasis [72]. This makes the tumor vessels more susceptible to leakage, allowing tumor cells to colonize and metastasize directly into the vessels without going through a complex invasive process. However, it is interesting to note that in diabetic patients, palmitic acid (PA) treatment can induce damage to endothelial cells to release mtDNA and activate the cGAS-STING-IRF3 pathway in the cytoplasm, leading to upregulation of macrophage stimulating 1 (MST1), inactivation of Yes-associated protein (YAP) and inhibition of angiogenesis [73]. This is contrary to the situation in tumors, and the specific reasons still need to be further explored.

In addition to affecting the tumor vasculature, mtDNA has been significantly associated with the epithelial–mesenchymal transition (EMT) process in tumor cells. EMT is a process in which the epithelial tumor cells lose their adhesion ability and cause the migration of mesenchymal cells to promote metastasis and drug resistance [74]. Tumor cells could activate the EMT process, undergoing significant cytoskeleton reconstruction and phenotypic changes. Tumor cells transform into the mesenchymal state, then secrete various extracellular matrix-degrading enzymes to promote their infiltration into blood vessels and lymphatic vessels [75]. Depletion of mtDNA has led to the loss of epithelial cell features and a mesenchymal phenotype being gained [76]. For example, in human prostate cancer cells (LNCaP) and breast cancer cells (MCF-7), deletion of mtDNA led to the activation of the RAF /MAPK signaling pathway. This process was also accompanied by the expression of transforming growth factor-beta (TGF-β) and type I TGF-beta receptor (TGF-βRI) [77]. All of these indications suggested that the tumor cells had acquired higher invasiveness [77]. Similarly, in breast cancer, a reduced mtDNA content in human mammary epithelial cells (HMECs) activated calcineurin-dependent mitochondrial retrograde signaling pathways that induced EMT-like reprogramming [78]. The TGF-β-EMT signal was activated in single breast tumor motile cells during this period, and was transferred to distal organs through hematogenous spread [79]. It is also noteworthy that the reduction in mtDNA also promoted the generation of breast cancer stem cells. Both these changes could promote the development of metastatic breast cancer [78]. On the contrary, the relatively high copy number of mtDNA was associated with low protein expression of E-cadherin and high expression of Vimentin in TE1 esophageal squamous cell carcinoma (ESCC) cells. The high mtDNA copy number appeared to contribute to the high bioenergetic function of mitochondria and provided favorable conditions for the invasion of esophageal cancer [80]. In colorectal cancer cells (SW620), the high expression of TFAM and mtDNA encoded ND6 and cytochrome c oxidase subunit II (COX-II), together with the high expression levels of the EMT markers N-cadherin and Vimentin, which may confer an advantage for the migration and invasion in CRCs [62]. EMT could be regulated by various signaling pathways, including TGF, HIF, Snail and Notch pathways [81]. It has been shown that TGF-β could convert mouse mammary epithelial NMUMG cells into an invasive fibroblastoid phenotype [82]. Subsequently, TGF-β has been proven to induce EMT in varieties cell lines, meaning it is considered to be a major EMT inducer. The inhibition of the TGF-β pathway in mesenchymal mouse colon carcinoma CT26 cell lines reduced invasion and metastatic formation in xenograft tumors [83]. The same phenomenon of EMT gene signal activation was be verified in diseases other than cancer. Aldosterone reduced the expression of PGC-1α to increase its acetylation and injure the mtDNA. It induced mitochondrial dysfunction and EMT to promote renal tubulointerstitial fibrosis [84] (Figure 3b). This also provided us with a new direction to prevent the metastasis of tumor cells through EMT.

### 3.3. mtDNA during Cancer Cell Survival in the Circulatory System

Tumor cells are shed from tumor tissue into the circulatory system for distant metastasis, but most of the scattered cells are not viable [85,86]. Disseminated cells are placed under extreme stress (from the immune system, ROS, hypoxia, hypotrophy and matrix changes), which greatly increases the pressure on their survival from one organ to another [6]. Studies have reported that the increased antioxidant capacity of tumor cells helps them survive after they shed from the tumor. In lung cancer mouse models, antioxidants can reduce oxidative stress and promote tumor progression [87]. Mutations in the mtDNA ATP synthase subunit 6 gene (MTATP6) can mediate resistance to apoptosis and promote survival in tumor cells [88]. When tumor cells were subjected to external stress or attack, mitochondrial released mtDNA to initiate their own protection programs to face the threats. At this point, a group of genes known as interferon stimulated gene (ISGs) came into play to protect their own nDNA, which was also the reason for the chemotherapy resistance of melanoma [37]. The mtDNA could also affect the level of p-glycoprotein (P-gp) and anti-apoptotic molecules such as Bcl-2, while P-gp is known to play an important role in mediating multi-drug resistance (MDR) in tumors [89,90]. Various evidence has indicated that aberrant mtDNA greatly enhanced the ability of tumor cells to cope with different threats, ensuring their survival in harsh environments and promoting metastasis. Hypoxia is widespread in tumor tissues [91], and overcoming hypoxic conditions is very important for tumor cell survival and metastasis [92]. Under hypoxic conditions, HMGB1 could mediate the binding of mtDNA and TLR9, thereby activating the protumorigenic signaling pathways to promote the growth and metastasis of hepatocellular carcinoma cells (HCC) [92]. In cancer cells with mutation in the mtDNA ND6 subunit G13997A, the expression of HIF1-α and MCL-1 was increased [19,93]. HIF1-α is one of the main regulators that adapt to hypoxia, and MCL-1 is an anti-apoptotic factor under hypoxia. Therefore, the high expression of these genes enabled tumor cells to survive under harsh conditions and effectively induce metastasis [94]. HIF-1 also regulates lactate dehydrogenase (LDH), monocarboxylate transporter (MCT) and other glycolytic enzymes, which is conducive to glycolytic conversion [95,96]. Furthermore, it can upregulate PDK, an inhibitor of pyruvate dehydrogenase (PDH), to inhibit the oxidative metabolism and promote metastasis [97]. Once tumor cells enter the circulatory system they became circulating tumor cells (CTCs), and they have to alter their metabolism in response to changes in environmental stress, especially hemodynamic shear stresses [98]. For example, compared to primary tumor cells, CTCs from breast cancer were mainly dependent on OXPHOS and increased ATP production. The transcriptional coactivator PGC-1α may play an important role in this. Overexpression of PGC-1α could promote the mitochondrial OXPHOS and enhance the invasion ability of breast cancer cells [99]. Some experiments have designed a variety of heterogeneous m.8993 T > G mutations in the mitochondrial gene MT-ATP6, which is called mTUNE line [100,101]. It has been shown that high levels of heterogeneity caused mTUNE cells to undergo glycolysis and metabolic conversion, while low levels of heterogeneity did not occur [102], suggesting that high heterogeneity levels of mtDNA mutations can promote tumor growth and metastasis.

During the various steps of metastasis, tumor cells are exposed to the immune system and recognized and killed by immune cells. However, tumors and their metastatic derivatives have developed strategies to overcome immune mechanisms. Tumor cells are able to induce the body to generate immunosuppressive cells and humoral suppressive factors, and are able to reprogram tumor-infiltrating immune cells to differentiate into immunosuppressive cells during metastasis [103]. CTCs can secrete LDH5 and cause ADAM10-mediated shedding of NKG2D ligand MICA/MICB, which prevents the identification and elimination of cells by NK-cell-mediated lysis [104]. Otherwise the immunosuppressive cells suppress the function of CD8^+^ T cells and NK cells by secreting cytokines such as EGF, TNF-α, CXCL12, IL-10 and IL-6, thereby ensuring the survival of tumor cells [105]. Previously, mtDNA was thought to affect inflammation and immune responses. It can activate the cGAS-STING pathway, triggering the innate immune system and eliciting the type I IFN response [38,39]. We generally assume that the activation of SING should enhance the anti-tumor immunity of the body. However, advanced tumor necrosis was found to release DAMP, while mitochondrial DAMP (including mtDNA) could activate neutrophils to generate neutrophilic extracellular traps (NETs) [106], which have been shown to promote cancer-related formation and are associated with accelerated metastasis [107]. The depletion of CD8^+^ T cells and NK cells could increase tumor metastasis without affecting the progression of the primary tumor [108]. CD8^+^ T cells restrict the metastatic growth of cancer cells from the primary tumor; when MERTK, a tyrosine kinase receptor that inhibits NK cells activation, is inhibited, NK cells have the ability to reject metastatic tumor cells [109,110]. Mitochondrial Lon protease (LON) is a chaperone and DNA-binding protein. When LON is overexpressed in oral cancer, oxidized mtDNA is released into the cytoplasm, which then induces interferon (IFN) signals through cGAS-STING-TBK1. This upregulates the expression of programmed cell death ligand 1 (PD-L1) and indoleamine2,3-dioxygenase1 (IDO-1), which inhibits the activation of T cells [111].

### 3.4. mtDNA in Cancer Cell Colonization

Tumor cells produce a variety of soluble factors that can induce the formation of a non-maturing pre-metastatic tumor microenvironment at new organ tissue sites or non-in situ tumor sites in the same organ. Micrometastasis foci are formed when tumor cells arrive and colonize in the pre-metastatic microenvironment. With the exception of tumor cells, various cell types mix in the tumor microenvironment, such as fibroblasts, adipocytes, endothelial cells and myocytes. In prostate cancer, mtDNA mutations in potentially bone-metastatic cells may preferentially alter the bone and bone matrix microenvironment. These mtDNA mutations increase the secretion and expression of fibroblast growth factor 1 (FGF-1) and focal adhesion kinase (FAK) to promote prostate cancer bone metastasis [112]. Tumor cells could recruit immunosuppressive cells such as myeloid-derived suppressor cells (MDSCs), tumor-associated macrophages (TAMs), regulatory T cells (Tregs), T helper 17 cells (Th17) and tumor-associated neutrophils (TANs) to the primary and secondary tumor sites, creating an immunosuppressive microenvironment. It has been reported that mtDNA stress in HCC cells with increased mitochondrial division significantly induced the increase in CCL2 production by activating the TLR9-mediated NF-κB signaling pathway. CCL2 could promote the recruitment and polarization of TAMs, consequently advancing the progression of HCC [113]. By secreting cytokines to promote the recruitment and polarization of TAM, HCC cells can induce the formation of the immunosuppressive microenvironment and promote tumor cell metastasis and colonization [114]. Analogously, IL-1β induced IL17 expression in γδ T cells, leading to systemic granulocyte colony-stimulating factor (G-CSF)-dependent expansion and neutrophil polarization, inhibiting the function of CD8^+^ T cells and promoting tumor metastasis. In addition to recruiting immunosuppressive cells, tumors can also regulate the function of immunosuppressive cells to enhance metastasis [115]. For instance, tumor-derived factors such as galectin-1 can promote systemic immunosuppression by regulating the clonal expansion and function of Treg, thereby enhancing breast cancer metastasis [116]. Additionally, tumor-infiltrating plasmacytoid dendritic cells (ρDCs) could increase MDSCs and Tregs in transplanted breast cancer tissues and reduce the cytotoxicity of CD8^+^ T cells to promote tumor bone metastasis [117] (Figure 3c). Immature pDC recognized the CpG sequence on mtDNA through TLR9 [118], and then induced the generation of CD4^+^CD25^+^Treg cells after activation and maturation. Treg cells secrete cytokines IL10 and TGF-β, which produce powerful immunosuppressive functions. We inferred that mtDNA exerts an immunosuppressive effect by enabling ρDC maturation. Inhibition of cytosolic DNA sensing is a strategy that tumor cells use for immune evasion [119]. The mtDNA induced by myocardial infarction could inhibit the secretion of IL-6 and TNF-α in granulocytes, which may account for the patients’ susceptibility to infection after injury or myocardial infarction [120]. The equal reaction may also occur in tumor patients. The mtDNA reduced the secretion of cytokines such as IL-6, decreasing the body’s anti-tumor immune capacity and promoting the progress of the tumor. Furthermore, fascin, a type of actin, promotes metastatic colonization in lung cancer by enhancing metabolic stress resistance and mitochondrial OXPHOS. Fascin was directly recruited to mitochondria in response to metabolic stress to stabilize mitochondrial actin filaments (mtF-actin). Mechanistically, Fascin and mtF-actin controlled the balance of mtDNA to promote mitochondrial OXPHOS. The results suggested that the dysregulated actin cytoskeleton in metastatic lung cancer could be targeted to realign mitochondrial metabolism and prevent metastatic recurrence [121].

## 4. Future Prospects

### 4.1. mtDNA as a Potential Indicator for Cancer Diagnosis

Furthermore, mtDNA affects the occurrence and progression of tumor. Large-scale data from the International Cancer Genome Consortium (ICGC) and the Cancer Genome Atlas (TCGA) have confirmed that approximately 60% of solid tumors have at least one mtDNA mutation [122,123,124]. Additionally, mtDNA is connected with the mitochondrial inner membrane, which is rich in lipids, and the relatively high ratio of fat to DNA in the mitochondria makes mtDNA extremely sensitive to liposoluble substances. As a result, lipophilic carcinogens often tend to choose mtDNA as their primary site for binding and attack. Compared to nDNA, carcinogens prefer mtDNA, causing mtDNA mutations [125]. Endogenous damage factors such as oxygen free radicals produced by tumor cells can easily attack mtDNA. Additionally, mtDNA is susceptible to mutation after being attacked, which in turn disturbs the respiratory chain and affects the normal mitochondrial oxidative phosphorylation system and metabolic reprogramming [10,13]. Furthermore, mtDNA mutations lead to respiratory chain disorders that can increase the level of ROS, which in turn generate new mtDNA mutations, forming a vicious cycle. When mtDNA mutations accumulate to a certain extent, they can trigger cancer and promote its metastasis and other malignant processes. Additionally, one of the pathogenesis mechanisms of cancer is the inhibition of cell apoptosis. Mitochondria can regulate cell apoptosis well, whereas mtDNA mutations can lead to mitochondrial dysfunction. Therefore, this can inhibit cell apoptosis to accelerate the formation of cancer. Furthermore, mtDNA mutations are considered to be an early event of tumorigenesis. In addition to mtDNA mutations, alterations in copy number, transcription and expression levels of mtDNA may also be important causes of cancer occurrence and metastasis. The alterations in the amount of mtDNA in tumor cells may be associated with a loss of mtDNA replication ability. The amounts of mtDNA vary in different tumors, and even at different stages of the same tumor. In breast cancer, mtDNA decreased during stage 0-II and increased during stage II-IV, suggesting that mtDNA can be used as an indicator for breast cancer risk assessment [126]. Some studies have found that changes in mtDNA can be used as an indicator to assess tumor aggressiveness, and mtDNA plays a unique role in the treatment of a variety of cancers [127]. Recently, some researchers have analyzed the mtDNA defects in triple-negative breast cancer (TNBC) and divided TNBC into different invasive subgroups [128]. Based on the differences in mtDNA in patient tumor cells, the risk of the patient could be better evaluated, thereby providing more appropriate treatment options for the patient [128]. When exposed to physical or biochemical damage, mitochondrial dynamics are changed, followed by the release of mtDNA, which randomly integrate with nDNA through the nuclear membrane. Accordingly, the stability of the nuclear genome and cellular energy metabolism is altered, causing malignant transformation of cells. Although there was not enough evidence to show a direct link between mtDNA and tumorigenesis or metastasis, all of the indications suggested that mtDNA is at least involved in the mechanism of tumorigenesis and development, and that it may be a potential indicator for early diagnosis of cancer, or even a marker for cancer metastasis; this still needs to be further explored. If we can summarize the characteristics of mtDNA mutation between tumorigenesis and metastasis, this may provide a great direction for the early diagnosis of cancer and the prediction of tumor aggressiveness.

### 4.2. mtDNA as a Therapeutic Target for Cancer

Surgery, radiotherapy and chemotherapy have always been referred to as the three major traditional treatments for cancer, although targeted molecular therapy is not a new term. The role of mtDNA in inflammation and innate immunity means it can influence the sensitivity of tumor cells to radiotherapy and chemotherapy. Changes in mtDNA itself may also lead to malignant changes in cells. HeLa cells in cervical cancer showed increased radiosensitivity after mtDNA deletion [129], while most of other cancer cells (e.g., lung cancer A549 cell) showed significant radioresistance after mtDNA deletion. Apoptosis is considered to be one of the major forms of radiotherapy, while cell lines lacking mtDNA have shown greater resistance to apoptosis, mediating their radioresistance. Nevertheless, the increased radiosensitivity of the HeLa cell line with mtDNA deletion may be due to its infection by human papillomavirus and subsequently the inactivated p53 gene [130]. Ionizing radiation could upregulate the mRNA and protein expression of TFAM, while inhibition of TFAM could increase the radiation sensitivity of tumor cells. Similarly, mtDNA has also been found to affect the sensitivity of tumor cells to chemotherapy. In prostate cancer, the chemotherapeutic drug BMD188 (a fatty-acid-containing hydroxamic acid) was also effective against multi-drug-resistant cancer cells, but its action was dependent on the mitochondrial respiratory chain to induce apoptosis, whereas tumor cells with defective mtDNA demonstrated significant resistance to BMD188 [131]. In MDA-MB-231 cells with mtDNA deletion, the amount and volume of mitochondria increased, the mitochondrial cristae showed air-like changes (vacuolation) and the sensitivity to chemotherapy drugs was decreased. NADH dehydrogenase subunit 4 (MT-ND4) mutations made cells resistant to paclitaxel carboplatin therapy [132]. Both radiotherapy and chemotherapy can disrupt the mitochondrial structure, leading to mutations or the release of mtDNA. This could affect the level of ROS and adjust the tolerance of cells to radiotherapy and chemotherapy. Using mtDNA as the therapeutic target to sensitize radiotherapy and chemotherapy or reverse the malignant transformation of tumors and inhibit tumor metastasis may be a new avenue for clinical treatment.

## 5. Conclusions

Tumor cells exhibit genetic instability and are prone to mutations, deletions and translocations. Extensive research has been conducted on changes in the nDNA of tumor cells, while the exploration of mtDNA changes has only been noticed in recent years. Although there is much evidence that mtDNA plays an important role in cancer, there is still much debate about mtDNA’s role in tumors. It is not yet clear whether it acts as a promoter, a bystander, a terminator or an accomplice in tumor progression. Furthermore, mtDNA may be able to affect different signal transduction mechanisms and changes in energy metabolism, and the production of ROS is involved in tumor development and progression. However, the specific role of mtDNA in cancer has not been discovered, and there are many complex links between mtDNA and cancer and its treatment. As an important organelle, mitochondrial participates in a variety of important physiological functions, such as energy generation and signal exchange. Additionally, mtDNA, the mitochondrial genome, plays a regulatory role in inter-mitochondrial, intra-cellular and inter-cellular communication. Due to the complexity and redundancy of the mitochondrial genome, it still needs to be further explored. In this review, we summarized the role of mtDNA in tumor metastasis and proposed certain directions for mtDNA research. With the deepening understanding of mtDNA, its mtDNA is becoming more promising. It is expected to be used as a cancer detection index and therapeutic target to improve the prognosis and treatment of patients.

## Abbreviation

BER	Base excision repair
CSCs	Cancer stem cells
CTCs	Circulating tumor cells
CXCL 12	C-X-C motif chemokine ligand 12
Cybrid	Cytoplasmic hybrid
ESCC	Esophageal squamous cell carcinoma
FAK	Focal adhesion kinase
FGF-1	Fibroblast growth factor 1
G-CSF	Granulocyte-colony-stimulating factor
GLUT 1	Glucose transporter 1
HCC	Hepatocellular carcinoma cells
HR	Homologous recombination
ICAM-1	Integrin/intercellular adhesion molecule-1
IDO-1	Indoleamine2,3-dioxygenase1
ISGs	Interferon-stimulated gene
LDH	Lactate dehydrogenase
MCT	Monocarboxylate transporter
MDR	Multidrug resistance
MMEJ	Microhomology-mediated end joining
MMP 9	Matrix metalloproteinase-9
MST 1	Macrophage-stimulating 1
mtDNA	Mitochondrial DNA
NETs	Neutrophilic extracellular traps
OXPHOS	Oxidative phosphorylation
PA	Palmitic acid
PDK	Pyruvate dehydrogenase kinase
PD-L1	Programmed cell death ligand 1
PI3K	Phosphatidylinositol 3-kinase
POLG	Polymerase g
TAMs	Tumor-associated macrophage
TANs	Tumor-associated neutrophils
Tregs	Regulatory T cells
TCA	Tricarboxylic acid
TFAM	Transposition factor A
TGF-β	Transforming growth factor-beta
VDAC	Voltage-dependent anion channel
VEGF	Vascular endothelial growth factor
YAP	Yes-associated protein
